# Emotions shape memory suppression in trait anxiety

**DOI:** 10.3389/fpsyg.2013.01001

**Published:** 2014-01-03

**Authors:** Tessa Marzi, Antonio Regina, Stefania Righi

**Affiliations:** Psychology Section, Department of Neuroscience, Psychology, Drug Research and Child Health, University of FlorenceFirenze, FI, Italy

**Keywords:** cognitive control, memory suppression, emotions, trait anxiety, Think/No-think paradigm

## Abstract

The question that motivated this study was to investigate the relation between trait anxiety, emotions and memory control. To this aim, memory suppression was explored in high and low trait anxiety individuals with the Think/No-think paradigm. After learning associations between neutral words and emotional scenes (negative, positive, and neutral), participants were shown a word and were requested either to think about the associated scene or to block it out from mind. Finally, in a test phase, participants were again shown each word and asked to recall the paired scene. The results show that memory control is influenced by high trait anxiety and emotions. Low trait anxiety individuals showed a memory suppression effect, whereas there was a lack of memory suppression in high trait anxious individuals, especially for emotionally negative scenes. Thus, we suggest that individuals with anxiety may have difficulty exerting cognitive control over memories with a negative valence. These findings provide evidence that memory suppression can be impaired by anxiety thus highlighting the crucial relation between cognitive control, emotions, and individual differences in regulating emotions.

## INTRODUCTION

It is probably wishful thinking that some experiences in life are best forgotten in order to protect our mental well-being. Thus, a fundamental question is whether it is really possible to intentionally suppress unwanted memories. This question is fascinating because the ability to exert cognitive control over memory has important implications for cognitive functioning and mental disorders (Banich et al., 2009; Depue, 2012). A still unresolved issue is whether we can inhibit the retrieval of specific emotional memories and control them. Cognitive control might be particularly important when we are confronted with memories that we wish to avoid thinking about, such as those that are emotionally negative. If these memories are not controlled, they might become intrusive in our consciousness (Erskine et al., 2007). This is especially the case of negative memories that have the capacity to unsettle our mental life by continuously intruding into cognitive operations. In some conditions, such as post-traumatic stress (PTSD) or anxiety disorders, the retrieval of these memories may cause serious distress and mental impairment (McNally, 2006; Schönfeld et al., 2007; Banich et al., 2009; Depue, 2012). Furthermore, persistent intrusive thoughts are a key source of distress across many forms of psychopathology (Magee et al., 2012).

The issue pursued in this study is part of this more general research topic on the complex interaction between cognitive control, memory, and emotions (for a review see Pessoa, 2008; Banich et al., 2009) with particular focus on individual differences in trait anxiety.

The core mechanisms underlying memory suppression are executive control processes that are engaged to control and inhibit the influence of unwanted memories by rendering retrieval more difficult (Anderson and Green, 2001; Levy and Anderson, 2008). The existence of this attempt to control memory has been demonstrated behaviorally and more recently also by means of neuroimaging and electrophysiological studies (for a review see Depue, 2011, 2012). These studies suggest that memory suppression occurs through cognitive inhibitory control as demonstrated by an increased activation of the prefrontal cortex which down-regulates the hippocampal regions. The prefrontal cortex is involved in sending an inhibitory signal to the target memory, stored in long-term memory, by disengaging episodic retrieval (Anderson et al., 2004; Depue et al., 2007; Benoit and Anderson, 2012; Depue, 2012).

A further aspect to consider in this complex interplay between cognitive control and memory is the role of emotions (Phelps, 2004; Pessoa, 2008, 2012; de Gelder and VandenBulcke, 2012; Van den Stock and de Gelder, 2012). Cognition and emotions are jointly associated and this is shown by the influence of emotions on perceptual processing, attention, executive functions, and memory (Pessoa, 2009; Tamietto and de Gelder, 2010; Pessoa et al., 2012). In this regard, memories for emotional events appear more vivid and are often better encoded and remembered than neutral events (Kensinger and Corkin, 2003). On one hand, suppressing memories with an emotional content might be harder due to the evidence that they are normally better represented and retrieved than are neutral experiences (LaBar and Cabeza, 2006) as is well known for intrusive memories in PTSD or anxiety (McNally, 2006). On the other, however, one should consider that emotional memories might be particularly suitable to motivate our cognitive control strategies to either promote remembering or hinder it (Pessoa, 2009; Levy and Anderson, 2012; van Schie et al., 2013). The findings on memory control over emotions are quite mixed: some studies found an enhanced suppression for negative emotions (Joormann et al., 2005; Depue et al., 2006; Lambert et al., 2010) suggesting that it is easier to forget negative memories while other studies reported smaller or no suppression effects for negative emotions (Marx et al., 2008; Murray et al., 2011).

The above studies have shown that it is possible to establish a relation between executive control of memory and emotions; however, a third relevant point to consider is why sometimes this control is missing or difficult to exert. In this vein, Levy and Anderson (2008) suggested that the absence of suppression might be due to individual differences in executive control. A particular condition in which executive control functions are not efficient as a result of negative emotions is anxiety (Eysenck et al., 2007; Banich et al., 2009). Specifically, individuals with anxiety may have difficulty exerting cognitive control over emotional information (Engels et al., 2007). It is well known that anxiety is characterized by a poorer executive control which might impair functions such as inhibition and attentional control (Bar-Haim et al., 2007; Eysenck et al., 2007; Derakshan et al., 2009; Righi et al., 2009; Ansari and Derakshan, 2011; Choi et al., 2012). There is compelling evidence that anxious individuals show increased attentional capture by cues signaling danger and are more likely to interpret emotionally ambiguous stimuli as threatening (Bishop, 2007).

Neurocognitive theories of anxiety have suggested that the amygdala activity is increased in anxiety in response to potential threat (Mathews et al., 1997). This hyperactivity could in turn strongly bias bottom–up attention allocation for threat (Bishop et al., 2004). Studies investigating the role of frontal cortical regions in the top–down control of emotion have shown that anxious individuals may have not only increased amygdala activity but also decreased recruitment of frontal control areas (Bishop et al., 2004; Bishop, 2009; Ferneyhough et al., 2013). It has been widely demonstrated that in high anxiety individuals threat-related processing is enhanced and the attempts at thought suppression generate an enhanced memory bias for threat material (Coles and Heimberg, 2002; Kircanski et al., 2008).

In the light of this evidence the aim that motivated this study was to explore the relation between memory suppression and emotions in high and low trait anxiety individuals. To do that we employed a specific paradigm that has enabled to demonstrate the ability to strategically forget information: the “Think/No-think” paradigm, developed by Anderson and Green (2001). This paradigm requires participants to associate pairs of stimuli, learn these pairs to a high degree thus establishing episodic long-term memory representations and gain control over the encoded memories by either recalling or inhibiting recall. In the present study this procedure was used with neutral words associated to emotional negative, positive, and neutral scenes. Success in this task likely relies on some active process that enables to stop the to-be-forgotten item from reaching consciousness (Anderson et al., 2004). Therefore, a crucial characteristic of this task relies on the ability to inhibit a learned item.

This paradigm has been previously widely used to uncover the neural underpinnings of memory suppression (e.g., Anderson and Green, 2001; Anderson et al., 2004; Joormann et al., 2005; Depue et al., 2006, 2007; Paz-Alonso et al., 2009, 2013; Anderson et al., 2011; Murray et al., 2011; Waldhauser et al., 2012; Depue et al., 2013; van Schie et al., 2013), although some studies failed to find a reliable suppression effect (see Bulevich et al., 2006).

Some relevant previous studies that employed the Think/No-think are noteworthy to mention. The study by Depue et al. (2006) showed that for both verbal and non-verbal material inhibitory influences of cognitive control were larger for negative than neutral items. Similar results were also found by van Schie et al. (2013), who suggested that direct suppression might impair negative stimuli. Furthermore, there is evidence that both emotional and neutral information can be successfully suppressed from memory (Murray et al., 2011). In similar vein, memory suppression was observed following the “no think” phase for memories associated with emotionally negative material (Lambert et al., 2010). In contrast to these studies, strongest inhibition effects were found also for highly arousing pleasant words (Marx et al., 2008).

However, only two studies have so far investigated this question considering anxiety, one by using neutral words (Waldhauser et al., 2010), and the other by using neutral and negative pictures (Dieler et al., 2013). The former found that better suppression was predicted by lower trait anxiety; the latter showed that higher ruminative tendencies and trait anxiety interfered with successful thought suppression, underlining the importance of considering personality characteristics in memory suppression tasks.

On the basis of this evidence, the present study was specifically focused on the interaction between emotion and cognitive control. Our hypothesis was to find differences in high compared to low anxious individuals in the retrieval of suppressed negative compared to neutral and positive memories. Since anxiety is characterized by a decreased executive control and by a bias for emotional events one would expect to find an enhanced difficulty in anxious individuals to control and suppress “unwanted” emotionally negative memories.

## MATERIALS AND METHODS

### PARTICIPANTS

Thirty right-handed participants (16 females; age 20–30) were selected from a sample of 150 university students based on their scores on the State-Trait Anxiety Inventory (STAI). Participants were matched for age, years of education, were native Italian speakers, had normal or corrected-to-normal vision and no neurological or psychiatric disorders. All participants gave informed consent and received class credit for taking part in the experiment that was carried out in accordance with the guidelines of the Declaration of Helsinki.

We subdivided our sample in two groups (*n* = 15 for each group) with high and low scores at the STAI (Spielberger et al., 1970), respectively. The STAI consists of 40 items measuring anxiety along the two distinct dimensions of state and trait. We used the Italian version of the STAI (Sanavio et al., 1997) that was also used previously by Righi et al. (2009); only scores obtained in the trait anxiety questionnaire were considered. Participants were classified as low-trait anxious or high-trait anxious if they scored one standard deviation below or above mean values, respectively (the normative scores considered were: for males up to 25 years old: Mean: 40.6, SD: 8.3; for males above 25 years: 37.8, 8.7; for females up to 25 years: 43.1, 9.3; for females 25 years: 42.2, 11).

### STIMULI

Stimuli consisted of 99 neutral words and emotional scenes (negative, positive, and neutral). The scenes were selected, on the basis of the normative ratings, from the International Affective Picture Series (IAPS; Lang et al., 1995) and matched for size and luminance. All words used were controlled for length, grammatical class, frequency of use, emotional valence, concreteness, and meaningfulness on the basis of a standardized database (Barca et al., 2002).

### EXPERIMENTAL PROCEDURE

**FIGURE 1:**
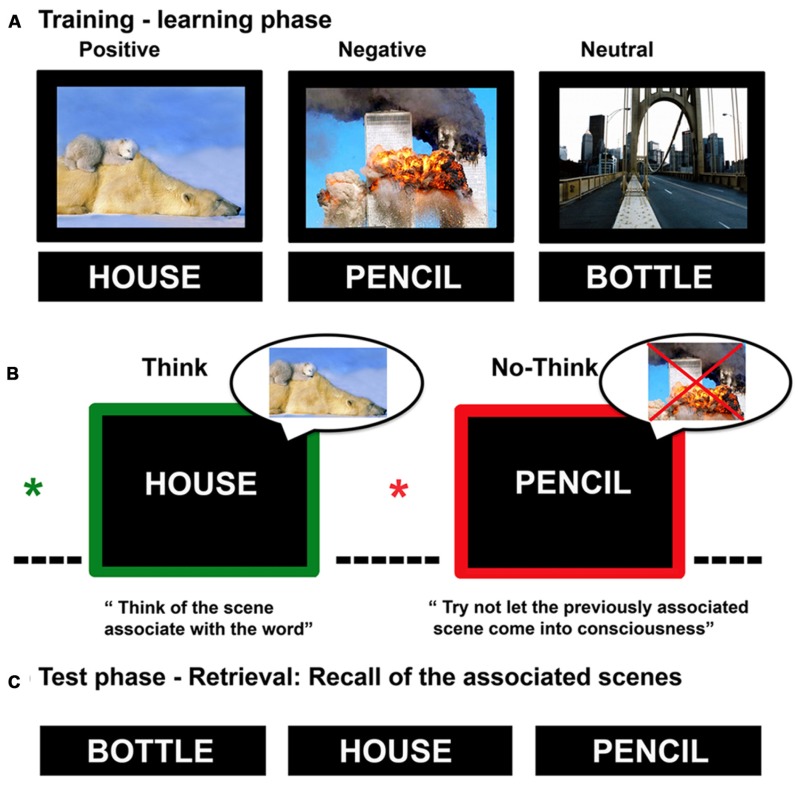
**Representation of the Think/No-Think procedure, divided in three phases: (A)** training-learning in which participants studied pairs of neutral words and unrelated emotional scenes, and were then tested to ensure learning to criterion; **(B)** only word were presented within a green (Think condition) or red (No-Think condition) frame and pre-cued by a green or red fixation (^*^); participants during “think” trials were instructed to try and recall the associated scene, whereas for “No-think” trials they were instructed to put the paired associate out of mind entirely. 45 pairs of stimuli were not presented during the Think/No-think phase because they served as baseline; **(C)** participants were asked to recall the scene associated with the presented word-cue. (Images of scenes were taken from IAPS, Lang et al., 1995).

The experimental session consisted of three blocks (33 trials for each block), each including a complete Think/No-think procedure characterized by: (1) training-learning phase, (2) Think/No-think phase, and (3) test-retrieval phase; the procedure is shown in **Figure 1**. A similar blocked design of the Think/No-think task was used in previous studies (Depue et al., 2006; Hanslmayr et al., 2009). The procedure was divided into the following phases:

(1) In the training-learning phase of each block, participants learned to remember 33 word-scene pairs (all the words were neutral in emotional valence while the scenes could either be positive, negative, or neutral), which were displayed on a screen for 4,000 ms. Participants were first instructed to learn all word-scene pairs, and then their memory was tested by showing only the word (cue) and asking to recall the related scene by verbally describing it. If they pressed the “I recall” button they were requested to verbally describe the scene by producing five words that described each picture. These descriptions were then scored as correct when all five words were congruent to the right scene or incorrect. This learning procedure was repeated until participants could recall the correct scene, previously paired with a word, with 70% accuracy over all pairs (an average of two repetitions).
(2) In the Think/No-Think phase, participants saw 18 words (for each block), half of these being assigned to the think condition and half to the no-think condition. In both conditions, a trial consisted of a fixation cross followed by the word-cue for 3,500 ms, and then by a 600 ms intertrial interval. The fixation cross turned, after a variable delay, from 1,000 to 1,500 ms duration, and was either red or green informing participants whether to prepare for thinking of a previously studied item (think: green) or to prepare to block it out (no-think: red). This was used to allow participants to prepare for the upcoming memory cue (Hanslmayr et al., 2009; Hanslmayr et al., 2010). As in Anderson and Green (2001), in the think condition, participants were instructed to: “Think of the scene previously associated with the word” whereas in the no-think condition, they were instructed to “Try not to let the previously associated scene come into consciousness.” Importantly, participants received direct suppression instructions by asking them to focus on the cue and suppress retrieval of target by blocking thoughts about it, without replacing it with other thoughts (see also van Schie et al., 2013). Within each condition (think, no-think), participants viewed all the words five times. The words not shown in this experimental phase (15 for each block with a total of 45 neutral words and 15 negative, 15 positive, and 15 neutral scenes) served as baseline (items never repeated).
(3) During the final test phase (for each block), participants were shown all the words they had seen previously (coming from think, No-think, and baseline conditions) and were requested to press one of two buttons if they could recall the associated scene. If they pressed the “I recall” button they were requested to verbally describe the scene by producing five words that described each picture. These descriptions were then scored as correct or incorrect. A correct response was scored when all five words were correct for each presented scene (these responses were matched with a pre-determined list of words that were relevant and congruent for each image).

The order of cue presentation was randomized so that the test order differed from the learning or experimental phases. Also the presented scenes and the presentation of the three blocks were randomized. A training session was carried out before the experiment started in order to get the participants familiar with the memory suppression task. All the participants reported, at the end of the experimental procedure, that they complied with the task instructions.

## RESULTS

### TRAINING-LEARNING PHASE

A repeated measure ANOVA factoring memory for blocks (first, second, and third), Emotion (neutral, negative, and positive), and Trait Anxiety (low and high) as between-factor was carried out on the accuracy of the training-learning phase. This specific analysis was performed to evaluate the effect of the sequence of blocks on memory performance in particular concerning the presence of differences between the two anxiety groups. The results showed that no main effect or interaction reached significance (*p*_s_ > 0.05). Furthermore, no significant differences emerged for the between Trait Anxiety factor (*p* = 0.43). The mean accuracy for each condition was: for the Low Trait Anxiety group: positive scenes: *M* = 89.90, SD = 13; negative scenes: *M* = 84.85, SD = 13; neutral scenes: *M* = 87.27, SD = 10; for the High Trait Anxiety group: positive scenes: *M* = 86.26, SD = 13.7; negative scenes: *M* = 86.87, SD = 13.3; neutral scenes: *M* = 87.56, SD = 13.2.

These analyses show that the groups do not differ for memory encoding capacity which in principle might have affected the observed results concerning memory suppression, see below. By the same token the results also show that there is no effect on memory performance as a function of the block sequence.

### RETRIEVAL

**FIGURE 2:**
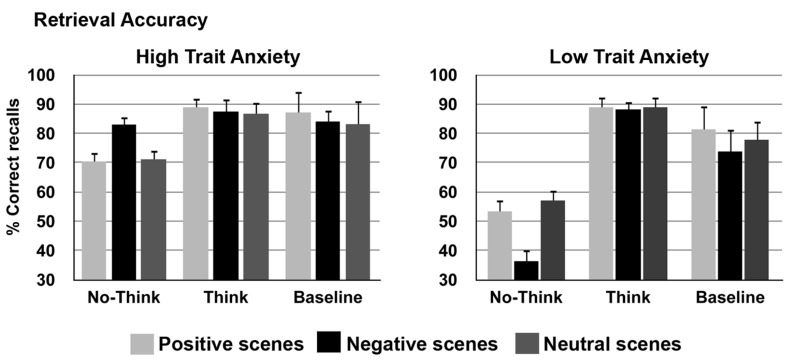
**Overall mean correct recall percentages of emotional scenes (positive, negative, and neutral) for baseline, think and no-think conditions, shown on the left for the high trait anxiety group and on the right for the low trait anxiety group**.

**FIGURE 3:**
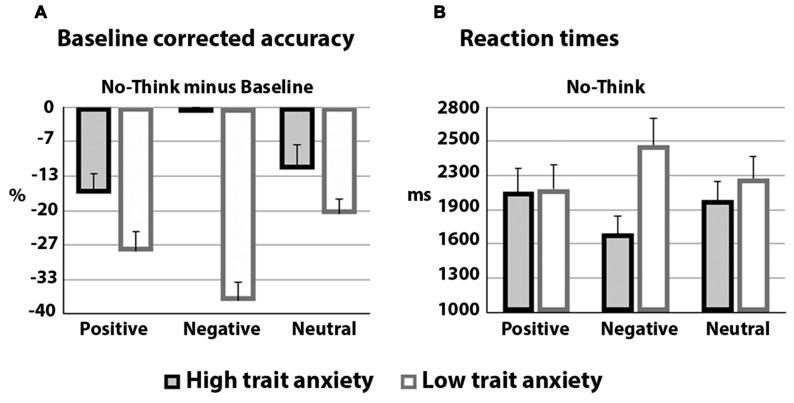
**(A)** Mean recall percentages, for the no-think condition, of emotional scenes (positive, negative, and neutral) as a function of low or high trait anxiety. **(B)** Mean reaction times for correctly recalled negative, positive or neutral scenes in the no-think condition, as a function of low or high trait anxiety.

A mixed repeated-measures ANOVA was performed on accuracy, percentage of correctly recalled scenes, during the test phase. Trait Anxiety (low and high) was the between-factor, Memory (think, no-think, and baseline), and Emotion (neutral, negative, and positive) were the within-subject factors. Accuracy scores for the final recall are plotted in **Figures 2 and 3A** shows baseline corrected accuracy for the No-Think condition (the baseline was subtracted from the No-Think condition). Bonferroni correction was applied for multiple comparisons. Additional ANOVAs were carried out for *post hoc* comparisons. No participant required more than three cycles of learning phase to achieve the learning criterion of 70%.

To exclude possible effects caused by the blocks order (first, second, or third presented block) a preliminary ANOVA was performed including as factor the Block order. No significant main effect or interaction emerged for this factor, (*p*_s_ > 0.05).

The analysis yielded a significant effect of Memory *F*(2,56) = 31.3, *p* < 0.001, $\eta_{p}^{2}$ = 0.53 showing higher percentage of recall after the think compared to both baseline and no-think conditions (both *p*_s_ = 0.001). Importantly, the memory suppression effect was demonstrated by the poorer accuracy for the no-think condition compared to baseline items, *p* = 0.001. This result replicates previous reports showing that direct suppression can impair recall of “unwanted” memories. However, most importantly for the purpose of the present study, this memory effects were modulated by individual differences in anxiety and emotions. This was reflected by the significant interactions Memory x Trait Anxiety, *F*(2,56) = 8.02, *p* = 0.006, $\eta_{p}^{2}$ = 0.22, and Memory × Emotion × Trait Anxiety *F*(4,112) = 3.4, *p* = 0.02, $\eta_{p}^{2}$ = 0.10.

*Post hoc* comparisons showed in the *low trait anxiety* group a poorer performance in recalling the scene for the no-think compared to the think, *p* < 0.001, and baseline conditions, *p* < 0.002; in addition think items were better retrieved than baseline items, *p* < 0.001. Low anxiety was related to a marginally significant effect of emotion, with poorer performance (that means, i.e., enhanced suppression) for negative compared to neutral scenes, *p* = 0.05. Overall, these results replicate the memory suppression effect in low anxiety individuals with a trend to better suppress negative scenes.

More importantly, the results showed that the memory suppression effect, with poorer recall in the no-think condition, was absent in *high trait anxiety* individuals who, in contrast, showed a higher accuracy for the no-think negative scenes compared to the low trait anxiety group, *F*(1,28) = 29.2, *p* < 0.001, $\eta_{p}^{2}$ = 0.51. Notably, this difference was related to the emotional value of the scenes. Specifically, emotionally negative scenes were less suppressed in high compared to low trait anxiety participants. Another difference was found by comparing the two groups: a higher percentage of recall for negative scenes in the baseline condition emerged for high compared to low trait anxious individuals, *F*(1,28) = 4.6, *p* = 0.04, $\eta_{p}^{2}$ = 0.14. In addition, in the high anxious group no significant differences emerged for the different memory conditions indicating an absence of memory suppression.

A further variable that has been considered was response latencies in recall (Reaction times: RT) for the emotional scenes. RTs for correctly recalled items were also submitted to repeated measures ANOVA (with the same factors as for accuracy), see **Figure 3B**; errors of omission (missing key press), and trials with RTs faster than 200 ms were excluded from the analyses.

The analysis showed two significant interactions: Memory x Trait Anxiety, *F*(2, 56) = 5.9, *p* = 0.007, $\eta_{p}^{2}$ = 0.17, and Memory × Emotion × Trait Anxiety, *F*(4,112) = 2.8, *p* = 0.043, $\eta_{p}^{2}$ = 0.09. The differences in RTs between the two groups were found for the No-Think condition. Paralleling the accuracy results, the high trait anxiety group was significantly faster, compared to the low trait anxiety group, in recalling negative scenes after the no-think instructions, *p* = 0.02. This shows that, for those items that were recalled correctly, time of response was influenced by the level of anxiety; high levels of trait anxiety hampered the memory suppression effect. No significant differences emerged for the high trait anxiety group while for the low trait anxiety group longer RTs were found for no-think compared to think items, *p* = 0.02.

### EVALUATION OF EMOTIONAL SCENES

**FIGURE 4:**
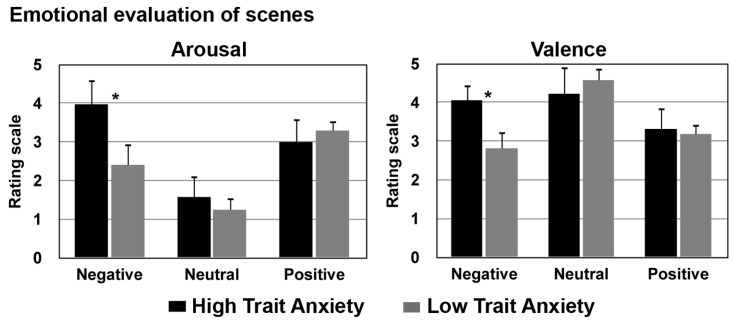
**Emotional rating of the presented scenes (on a 5-point scale: from not at all to very much).** Left panel: evaluation of arousal (how much was the scene considered arousing) for both groups; right panel: evaluation of valence: how much the presented scene was negative, positive, or neutral for low and high trait anxiety individuals (*p < 0.01).

To assess the participants’ subjective emotional response to valence and arousal, at the end of the Think/No-think paradigm all scenes were evaluated first for valence and second for arousal. Participants were instructed to recognize the presented scenes as negative, positive, or neutral and then to rate them on a 5-point scale, ranging from “not at all” to “very much,” first for valence and then for arousal. These ratings were analyzed with repeated measures ANOVAs with Trait Anxiety (low and high) as between-factor and Emotion (negative, neutral, and positive) as within factor; the results are reported in **Figure 4**.

### VALENCE

In this rating negative, positive, and neutral scenes were presented and participants were asked to rate how much emotional they found the scene (different questions were presented for each emotion; for negative scenes: “how much, from 0 to 5 points, is this scene negative for you?”; for positive scenes: “how much, from 0 to 5 points, is this scene positive for you?”; for neutral scenes: “how much, from 0 to 5 points, is this scene neutral for you?”).

Emotion and Trait Anxiety were significant, [*F*(2,56) = 65.1, *p* < 0.001, $\eta_{p}^{2}$ = 0.69; *F*(1,28) = 9.01, *p* = 0.006, $\eta_{p}^{2}$ = 0.24]. These effects were further qualified by the significant interaction Emotion by Trait Anxiety, *F*(2,56) = 27.7, *p* < 0.001, $\eta_{p}^{2}$ = 0.51. *Post hoc* comparisons showed that negative scenes were judged as more negative by high compared to low trait anxiety individuals, *p* < 0.001, $\eta_{p}^{2}$ = 0.7. Furthermore, in the high trait anxiety group positive scenes obtained lower ratings compared to negative, *p* < 0.001, and neutral scenes, *p* = 0.001; in the low trait anxiety group neutral scenes were rated with higher scores compared to positive and negative scenes, *p*_s_ < 0.001.

### AROUSAL

The ANOVA showed a significant effect of Emotion and of Trait Anxiety, [*F*(2,56) = 186.7, *p* < 0.001, $\eta_{p}^{2}$ = 0.87; *F*(1,28) = 15.4, *p* < 0.001, $\eta_{p}^{2}$ = 0.35]. These effects were further characterized by the significant interaction Emotion by Trait Anxiety, *F*(2,56) = 40.4, *p* = 0.001, $\eta_{p}^{2}$ = 0.59, showing that negative scenes were evaluated as more arousing by the high compared to the low trait anxiety group, *p* = 0.001, $\eta_{p}^{2}$ = 0.64. Furthermore in the anxious group negative scenes were considered more arousing than neutral and positive scenes, *p*_s_ < 0.001 while a different response pattern emerged in the low trait anxiety group with positive scenes evaluated as more arousing compared to neutral and negative scenes, *p*_s_ < 0.001.

These results confirmed that there was a threat bias specific for negative scenes in individuals with high trait anxiety.

## DISCUSSION

The present study examined whether memories for emotional scenes could be forgotten, by considering individual differences in trait anxiety in the Think/No-think paradigm.

We confirmed previous work by showing that the suppression of previously encoded target scenes can cause later forgetting of unwanted memories (Anderson and Green, 2001; Anderson et al., 2004; Depue et al., 2006, 2007; Levy and Anderson, 2008) but that this suppression effect is highly influenced by trait anxiety. Importantly, the present findings suggest that higher levels of trait anxiety interfere with and disrupt memory control by causing a lack of intentional suppression of memories. These results are in keeping with a recent study showing that high levels of trait anxiety predict the ineffectiveness of memory suppression for neutral words (Waldhauser et al., 2010). Furthermore, the importance of considering individual differences in the ability to suppress thoughts was very recently shown by Dieler et al. (2013), who, by employing psychometric measures, demonstrated the influence of certain depressive and anxious traits on thought suppression.

Importantly, here we show that this difficulty of high anxiety individuals in controlling memory emerged especially in the recall of emotionally negative rather than positive or neutral scenes. These findings highlight the strength and importance of the relation between executive control and emotions. The low trait anxiety group showed the classical memory suppression effect expected in the Think/No-think task with a better recall for “think” scenes and a poorer recall for “no-think” items compared to both the think and the baseline conditions demonstrating that the control over memory occurred successfully (Anderson and Green, 2001; Levy and Anderson, 2002). Moreover, the suppression effect on accuracy was also paralleled by the longer-latency responses for items recollected after the no-think condition. The difference found between no-think and baseline items, with a poorer recall for the former, known as the “negative control effect,” shows that retrieval suppression causes more forgetting than would normally occur after a period of time; this was reported in several previous studies using words (Anderson and Green, 2001; Anderson et al., 2004; Paz-Alonso et al., 2009; van Schie et al., 2013), faces, and scenes (Depue et al., 2006, 2007).

Interestingly, in the present study this pattern of results was not found in high trait anxiety individuals that showed no differences between think, no-think, and baseline conditions and had a better recall with respect to non-anxious individuals, for no-think negative scenes. Negative scenes coming from the no-think procedure showed enhanced accuracy and shorter reaction times in the high compared to the low trait anxiety group. One should note that these results are unlikely to be related to the use of different strategies causing possible interferences during the no-think phase because participants were strongly discouraged from generating other memory associations while viewing the word-cues (Bäuml and Hanslmayr, 2010; van Schie et al., 2013).

Based on these results we suggest that it is possible to control various aspects of memory by engaging cognitive mechanisms of control and inhibition but that these operations become dysfunctional with increasing levels of anxiety (Eysenck et al., 2007). Thus, in this case, anxious traits might represent a marker of inefficient cognitive control functions (Dieler et al., 2013). In keeping with that, behavioral results indicated that individuals with Attention Deficit Hyperactivity Disorder showed no reductions in recall during no-think trials suggesting an inhibitory deficit (Depue et al., 2010); moreover, encoding and retrieval inhibition in directed forgetting were impaired in individuals with obsessive compulsive disorder (OCD; Konishi et al., 2011), and also depressed participants have been reported to be impaired in suppression performance (Hertel and Gerstle, 2003; Joormann et al., 2005; Hertel and Mahan, 2008).

Moreover, the absence of intentional suppression was more evident in anxiety when participants were confronted with memories that had a negative value (see also Dieler et al., 2013). In line with this result, the PFC was found to be less activated during the processing of threat-related stimuli in participants with high STAI compared to those with low scores (Bishop et al., 2004). In relation to the role of emotions in memory suppression, Depue et al. (2006) showed a greater no-think effect for negative than for neutral valence, suggesting that negative items may be easier to suppress. Moreover, suppression was observed for negative, but not for positive, emotional content by Lambert et al. (2010). Negative valences images have been shown to elicit greater cognitive control than neutral stimuli (Depue et al., 2006; Butler and James, 2010).

In line with these results, in the present study low trait anxiety individuals showed a tendency to be facilitated in the memory suppression of negative scenes. However, not all studies showed suppression for negative events, for example, using the Think/No-think task, highly arousing negative words were found to be resistant to memory suppression (Marx et al., 2008); and in a successful directed forgetting task memory control was found for neutral but not negative pictures (Hauswald et al., 2011). These differences might be related to the different tasks (Think/No-think or Directed forgetting) or stimuli used (scenes versus words). Furthermore also differences in the Think/No-think procedure might explain the discrepant results. In this regard, in the present study direct suppression instructions were given to the participants during the think/no-think phase and the results are consistent with recent work that used this suppression strategy (Bergström et al., 2009; Hanslmayr et al., 2009, 2010; Benoit and Anderson, 2012; van Schie et al., 2013). Accordingly, it has been recently shown that emotionally negative memories can be forgotten by direct suppression instructions during the Think/no-think phase (van Schie et al., 2013). Moreover, it has been shown that motivated expressive suppression in emotion regulation was associated with less anxiety (Egloff et al., 2006).

We believe that individuals with higher anxiety may lack the cognitive control mechanisms that allow the modulation of emotional memories. The effect of threat on executive functions has been recently reported showing that emotional stimuli can interfere with the resources underlying cognitive control (Lindström and Bohlin, 2012).

Another possibility is that high trait anxious individuals might be very sensitive to threatening stimuli and as a result, the encoding of emotional information might be so enhanced that normal cognitive control mechanisms become ineffective in modulating retrieval (Depue et al., 2006; Marx et al., 2008). This possibility might be reflected by the higher recall accuracy for negative scenes, that we found, in the baseline condition in the high compared to low trait anxiety group. In this regard, it is noteworthy to mention that, in the present study, high compared to low trait anxious individuals evaluated negative scenes as more negative and more arousing, confirming an enhanced sensitivity for negative emotions (Ewbank et al., 2009). It is possible that high trait anxious individuals are more captured by cues that trigger negative emotions and are more likely to interpret emotional stimuli as more threat-related than non-anxious individuals (Bishop, 2007). In this regard, it is well known that individuals who cannot effectively manage normally their emotional responses to everyday events experience longer and more severe periods of distress that may evolve into depression or anxiety (Mennin et al., 2007; Aldao et al., 2010). It is noteworthy to mention that during states related to anxiety glucocorticoids are generally elevated and the arousal produced by this effect might enhance memory for fearful events (for a review see Erickson et al., 2003). In this vein, there is evidence that emotion, learning, and memory might be affected by corticosterone receptors (Roozendaal et al., 1996).

Furthermore, anxiety might be viewed as the result of difficulties in regulating emotions and in using maladaptive strategies to cope with stressful life events (Mennin and Fresco, 2009; Aldao et al., 2010). It has been shown that emotional reactivity was associated with maladaptive coping strategies that are strictly linked to intrusive memories (Regambal and Alden, 2009).

The present findings might also be related to evidence suggesting that attempts to keep intrusive thoughts out of mind – through “thought suppression” – can unintentionally heighten the recurrence of intrusive thoughts particularly among anxious and depressed individuals (Wenzlaff and Wegner, 2000; Abramowitz et al., 2001). This line of research started with the classical study by Wegner et al. (1987) that found that participants attempting to suppress thoughts of a white bear ironically later experienced more white bear thoughts than participants not instructed to suppress. It might be that this happens especially when an individual with high trait anxiety attempts to suppress a thought, and specifically a negative thought that is more likely to become recurrent than for a person with low trait anxiety (Magee and Zinbarg, 2007). Thus, it is possible to link lack of suppression to persistence of intrusive thoughts especially among individuals with psychopathology or high trait anxiety (Magee et al., 2012). This is in accord with the difficulty in suppression found for high trait anxious individuals in the present study.

Our findings may be viewed as showing that deliberate avoidance of thoughts associated with an emotionally negative experience may lead to loss of memory (Lambert et al., 2010) but that this effect is disrupted when the executive control functions are not so efficient, as is the case in anxiety (Eysenck and Derakshan, 2011). These results, although taken cautiously, might have some implications for disorders in which ruminative thought and negative memories are dysfunctional, such as anxiety, depression, OCD, acute stress disorder (ASD; Aldao et al., 2010; Depue, 2011).

It is important to underline that memory suppression can be influenced by individual differences in the capacity for mnemonic control as shown also in a very recent study in which individuals who were able to suppress memory retrieval exhibited tighter coupling in the prefrontal cortex-cingulate-parietal-hippocampal network than individuals who did not (Paz-Alonso et al., 2013). It is likely that differences in the ability to block and control intrusive memories arise, in part, from differences in executive control abilities specifically in engaging inhibitory mechanisms (Levy and Anderson, 2008). fMRI and EEG studies converge on the view that retrieval success is voluntarily controlled during suppression trials (Bäuml and Hanslmayr, 2010) by showing a down-regulation of memory-related neural activity during no-think trials (Depue et al., 2007, 2013; Hanslmayr et al., 2009). It has been shown that voluntary suppression elicits increased activity in prefrontal areas and decreased activity in brain regions processing memory representations (Anderson et al., 2004; Depue et al., 2007) with the hippocampal volume associated to inhibitory processes of memory retrieval (Depue and Banich, 2012). This suggests that the PFC inhibits the neural mechanisms underlying memory processing mediating later forgetting of the suppressed information. Moreover, two electrophysiological effects were found that might reflect voluntary memory suppression, namely, an early anticipatory and a later item-directed process (Hanslmayr et al., 2009; Depue et al., 2013). Importantly, it has been shown that enhanced hippocampal activation occurs during the suppression of emotionally negative stimuli together with the activation of amygdala, anterior cingulate, and fusiform gyrus (Butler and James, 2010). Thus, during attempts to suppress negative memories regions involved in the emotional content of memory are activated, along with regions reflecting conscious recall (Butler and James, 2010).

In the light of this evidence we suggest that memory control and suppression are influenced by individual differences in trait anxiety as a result of a possible impairment in the efficiency of executive functions and specifically in inhibition processes (Eysenck et al., 2007; Righi et al., 2009; Eysenck and Derakshan, 2011).

We acknowledge several possible limitations of our study: first, the data cannot provide a detailed characterization of how recall for think and no-think trials varies as a function of repetition. Moreover, including a clinical sample might have lead to stronger conclusions and to possible implications for treatment. In relation to this point, it is noteworthy to report that training depressed participants to suppress negative material, by providing them with a suppression strategy, increased intentional forgetting of negative material (Joormann et al., 2009). Apart from these limitations, our results extend previous findings (e.g., Anderson and Green, 2001; Levy and Anderson, 2002; Depue et al., 2006) by demonstrating that the think/no-think paradigm is robust against variations of type of stimulus, number of repetitions, participant’s strategies, and emotional content of the stimuli.

In closing, the present study raises the possibility that cognitive control for negative valence memories, as reflected by the memory suppression effect, are impaired in high trait anxiety. Moreover, it highlights the importance of considering individual differences in memory suppressions studies. Just as each person has a unique fingerprint or face each of us has a unique emotional profile and a different way of responding to emotional events or memories.

This line of research needs to be further pursued, especially by testing clinical samples, to better uncover the complex interaction between cognitive control functions, memory encoding, retrieval, and emotions.

## Conflict of Interest Statement

The authors declare that the research was conducted in the absence of any commercial or financial relationships that could be construed as a potential conflict of interest.
